# Remedies and Their Uses

**Published:** 1907-03-30

**Authors:** 


### REMEDIES AND THEIR USES.

### Organic Arsenic Compounds.

*Arsenoferratose* is an organic preparation on the same lines as Ferratose, containing .3 per cent. Fe. and .003 per cent. As., both combined with protein. A tablespoonful therefore, in addition to the same amount of iron as is found in ferratose, will contain .0075 grain arsenic, making a daily dosage of .032 grain with 2.3 grains iron. The arsenic cannot be recovered from its compound by ordinary precipitants until the protein portion of the molecule has been digested and broken down. This preparation is manufactured by Bœhringer and Sons.

*Metharsinate.*—This preparation is closely related chemically to cacodylic acid and the cacodylates, which have been shown experimentally to have a remarkable influence on the blood. It is a white crystalline salt, soluble in water. It contains 45 per cent. by weight of arsenious acid. Its structure can easily be seen from the following formulæ: $H_3AsO_4$, arsenic acid may be written—

$$O{=}As\begin{cases}OH\\OH\\OH\end{cases}$$

If two of the hydroxyl (OH) groups are substituted with methyl ($CH_3$), cacodylic acid results—

$$O{=}As\begin{cases}CH_3\\CH_3\\OH\end{cases}$$

If *one* hydroxyl group only is changed, and the two hydrogen atoms remaining are combined with sodium, the disodium salt of monomethyl arsenic acid is produced—

$$O{=}As\begin{cases}CH_3\\ONa\\ONa\end{cases}$$

This, as prepared in a pure form, free from arsenite or arseniate of sodium, by Messrs. Clin and Co., is known by the trade name of metharsenate. The advantages of this preparation are that, unlike the cacodylate of sodium, it does not give to the breath and excretions the unpleasant odour of garlic, and it is well tolerated by the stomach. In ordinary doses it increases the blood-pressure and the formation of red blood-cells and hæmoglobin. It is said by Gautier to have a favourable influence on nitrogenous metabolism, and it tends in cases of tuberculosis to check the excessive oxidation processes which have been observed to occur. Under its influence pyrexia is diminished, and appetite and digestion are improved. It is also useful in checking the excessive vomiting sometimes so troublesome in pregnancy. In malaria, pernicious anæmia, and other hæmic diseases it gives good results. In skin diseases methylarsenate of sodium is valuable when the prolonged action of arsenic is required, as, unlike the cacodylate, it has never been observed to irritate the stomach. Owing to its slight toxicity it may be given in comparatively large doses. Two to three grains per day of the pure drug have been tolerated, but these are maximum doses. In tuberculosis, to check the pyrexial symptoms one-third to half a grain in divided doses is usually recommended, and in other cases (blood diseases) three-quarters of a grain would be considered an average dose. It is best given for four or five days, and then omitted for a like period, after which it is again administered. It has the great advantage over the cacodylates that it is suitable for administration by the mouth, and need not be given hypodermically. Pilules containing 0.1 grain of the drug, the dose being one to five during the day, are prepared by Messrs. Clin; and also a solution, the dose being five to twenty-five minims during the day. There is also a solution in sterilised tubes for hypodermic use.

